# Lowering β-Amyloid Levels Rescues Learning and Memory in a Down Syndrome Mouse Model

**DOI:** 10.1371/journal.pone.0010943

**Published:** 2010-06-03

**Authors:** William J. Netzer, Craig Powell, Yi Nong, Jacqueline Blundell, Lili Wong, Karen Duff, Marc Flajolet, Paul Greengard

**Affiliations:** 1 Laboratory of Molecular and Cellular Neuroscience, The Rockefeller University, New York, New York, United States of America; 2 Departments of Neurology and Psychiatry, University of Texas Southwestern Medical Center at Dallas, Dallas, Texas, United States of America; 3 Department of Psychiatry, Columbia University Medical Center, New York, New York, United States of America; Mental Health Research Institute and the University of Melbourne of Victoria, Australia

## Abstract

β-amyloid levels are elevated in Down syndrome (DS) patients throughout life and are believed to cause Alzheimer's disease (AD) in adult members of this population. However, it is not known if β-amyloid contributes to intellectual disability in younger individuals. We used a γ-secretase inhibitor to lower β-amyloid levels in young mice that model DS. This treatment corrected learning deficits characteristic of these mice, suggesting that β-amyloid-lowering therapies might improve cognitive function in young DS patients.

## Introduction

Down syndrome (DS) is a complex genetic disorder that includes varying degrees of intellectual disability [1], [2]. Occurring in approximately 1 in 700 births, DS results from trisomy of all or part of human chromosome 21 (trisomy 21) [3], which generally accounts for triplication of at least 100 genes. Among these is the gene encoding APP, as well as genes that upregulate APP expression [4]. Sequential cleavage of APP by β-secretase (BACE 1) and γ-secretase produces peptides of varying lengths (mainly 40 and 42 amino acids), collectively termed β-amyloid, or Aβ [5], [6], which is widely believed to be an acute mediator of cognitive impairment [7], as well as a causative factor in Alzheimer's disease (AD). Aβ is over-produced in Down syndrome patients throughout life. Indeed, Aβ serum levels are 200% to 300% higher than in karyotypically normal individuals, and Aβ accumulates within neurons and in amyloid plaques in juvenile and adult DS patients, respectively [8].

Increased Aβ levels in DS are believed to be responsible for the near universal prevalence of AD in adult DS patients. By age 40, most if not all DS patients have extensive amyloid plaque pathology and degeneration of basal forebrain cholinergic neurons, both characteristic features of AD, and most of these individuals develop AD dementia by the fifth and sixth decades of life [9]. However, it is not known whether elevated Aβ levels in DS children affect intellectual disability. To explore this possibility, we utilized the Ts65Dn mouse, which is widely considered the gold standard of Down syndrome mouse models [10]. Ts65Dn is characterized by partial trisomy of mouse chromosome 16, which contains genes homologous to the human chromosome 21 genes that are most consistently triplicated in DS, including three copies of *APP*, the gene encoding the amyloid precursor protein [11]. These mice exhibit pronounced cognitive deficits as early as 2–3 months of age [12], as well as other correlates of DS. Additionally, by 6 months of age, Ts65Dn mice begin a progressive, age-related decline in choline acetyltransferase (ChAT) levels and cognitive function [13], features that are common to adult DS and AD patients [14].

## Results

### DAPT alters levels of APP metabolites in 4-month-old Ts65Dn mice

**Figure 1:**
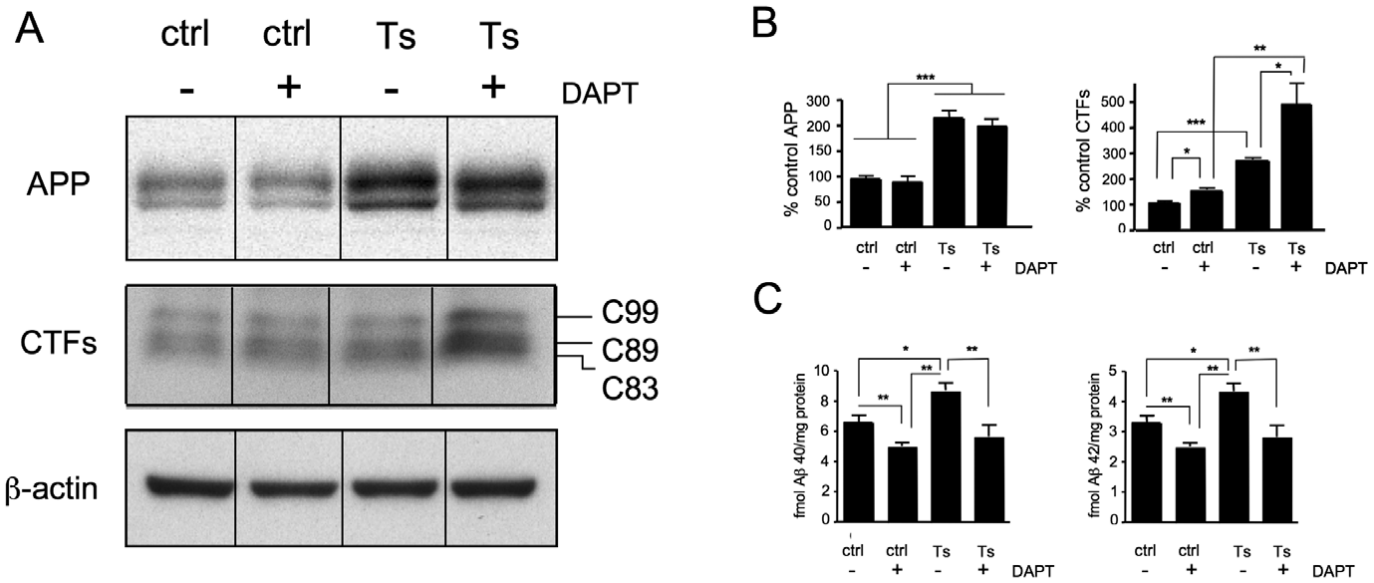
DAPT raises APP-CTF levels and lowers Aβ levels in brains of 4-month-old Ts65Dn mice. Four-month-old Ts65Dn mice and wild type colony mate controls were treated with vehicle or DAPT (100 mg/kg/day) for 4 days. (A) Representative western blots of APP, CTFs and β-actin from control (ctrl) and Ts65Dn (Ts) mice. (B) Left panel, quantification of APP (Students t-test, mean±s.e.m.,unpaired, two-tailed, n = 8 per group). Ctrl+Vehicle vs. Ts+Vehicle, p = 0.0003; Ctrl+DAPT vs. Ts+DAPT, p = 0.0002; Ctrl+Vehicle vs. Ts+DAPT, p = 0.0001; Ctrl+DAPT vs. Ts+Vehicle, p = 0.0006. Right panel, combined (C99, C89 and C83) CTFs (all means differ significantly, n = 8, 1-way ANOVA, p = 0.0002; significant differences between individual pairs of mean calculated by Students t-test, mean±s.e.m., unpaired, two-tailed). (C) Aβ40 and Aβ42 quantification from control and Ts65Dn mice. Left panel, Aβ40 (Students t-test, mean±s.e.m., unpaired, two-tailed, n = 6 per group); Ctrl+Vehicle vs. Ts+Vehicle, p = 0.0173; Ctrl+Vehicle vs. Ctrl+DAPT, p = 0.0043; Ctrl+DAPT vs. Ts+Vehicle, p = 0.0079; Ts+Vehicle vs. Ts+DAPT, p = 0.0082. Right panel, Aβ42 (Students t-test, mean±s.e.m., unpaired, two-tailed, n = 6 per group); Ctrl+Vehicle vs. Ts+Vehicle, p = 0.0169; Ctrl+DAPT vs. Ts+Vehicle, p = 0.0003; Ts+Vehicle vs. Ts+DAPT, p = 0.0052.

We used 4-month-old Ts65Dn mice and their disomic, colony-mates as controls. We compared APP levels, the β-secretase and α-secretase cleaved APP C-terminal fragments (C99, C89 and C83) [15], and Aβ40 and Aβ42 from hemibrains (lacking cerebellum) of mice that had been treated either with the γ-secretase inhibitor, DAPT [16], or vehicle for four days. Western blot analysis revealed that APP levels in vehicle-treated Ts65Dn mice were elevated to 225% of vehicle-treated controls (Fig. 1a, b), in agreement with some [17] but not all previous studies [18]. Levels of the β-secretase and α-secretase cleaved APP C-terminal fragments (C99, C89 and C83) in vehicle-treated Ts65Dn mice were elevated to 260% of vehicle-treated controls (Fig. 1a, b) suggesting that increased levels of Aβ might occur as a result of an enlarged precursor pool. Aβ40 and Aβ42 concentrations were elevated in vehicle-treated Ts65Dn mice to 132% and 139% of vehicle-treated controls, respectively (Fig. 1c).

DAPT treatment lowered Aβ40 and Aβ42 concentrations in Ts65Dn mice to 65% and 64% of vehicle-treated Ts65Dn mice, respectively (Fig. 1c). DAPT also decreased Aβ40 levels in control mice to 76% of vehicle-treated controls (Fig. 1c, left panel) and elevated APP C-terminal fragments to 149% and 180% of vehicle-treated control and vehicle-treated Ts65Dn mice, respectively (Fig. 1b).

### DAPT reverses Ts65Dn Cognitive Deficits in the Morris Water Maze

Ts65Dn mice are characterized by deficits in spatial learning and memory in behavioral tests, including the Morris water maze [19]. Similar cognitive deficits have been described for AD model mice. Since acute treatment with DAPT had previously been shown to rescue cognitive deficits in an AD mouse model [20], we tested the ability of DAPT to improve cognitive function in Ts65Dn mice.

**Figure 2:**
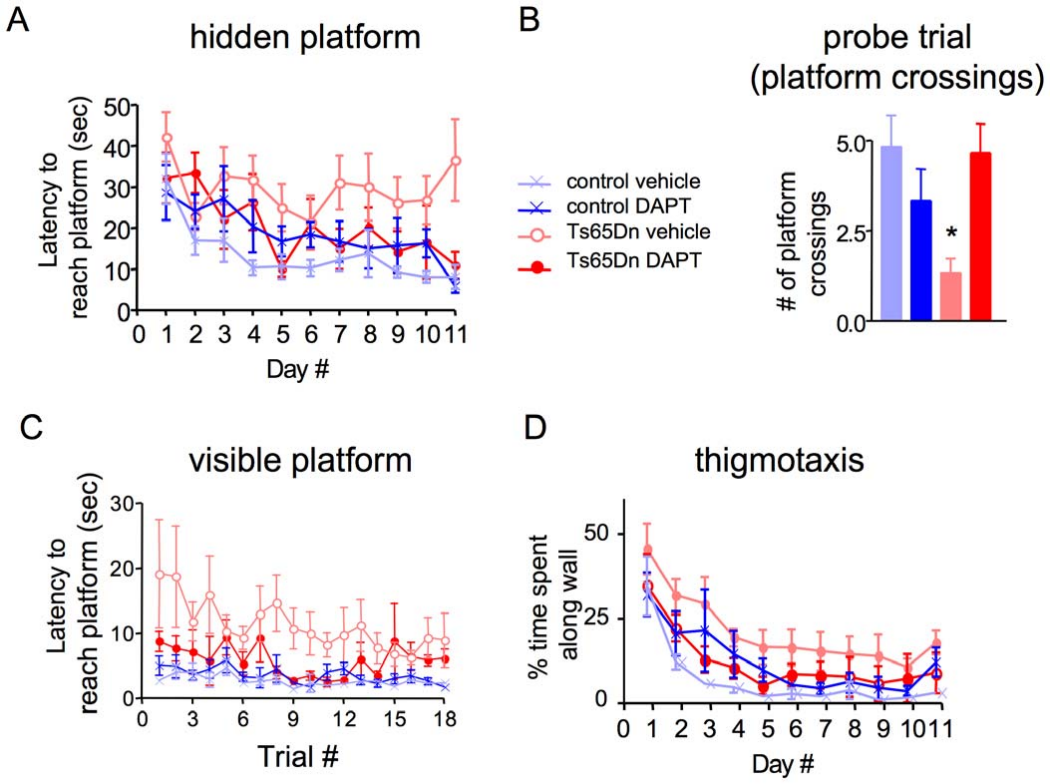
DAPT reverses cognitive deficits in 4-month-old Ts65Dn mice in the Morris water maze. DAPT was administered to Ts65Dn and control mice (100 mg/kg/day) two days prior to, and throughout, the maze testing. (A) Hidden platform test, latency to reach platform during training. (B) Probe trial on day 12, number of platform crossings. (C) Visible platform test, latency to reach platform. (D) Thigmotaxis. Statistical Analysis: n = 6 for all groups (A–D). (A) 2-way ANOVA with repeated measures revealed a main effect of genotype F1,20 = 11.31, p = 0.003 & Day F10,200 = 4.90, p = 3.00E-06 and an interaction between genotype and DAPT F1,20 = 7.73, p = 0.012. Post-hoc planned comparison test between Ts65Dn+vehicle and all 3 other groups (Ts65Dn+vehicle vs. Ts65Dn+DAPT p = 0.02, Ts65Dn+vehicle vs. control+vehicle p = 0.0003, Ts65Dn+vehicle vs. control+DAPT p = 0.008, n = 6 in all groups for all figures). (B) 2-way ANOVA for number of target platform crossings revealed an interaction between genotype and DAPT F1,20 = 8.46, p = 0.009. Post-hoc planned comparison test revealed a significant difference between Ts65Dn+vehicle vs. Ts65Dn+DAPT p = 0.01 and between Ts65Dn+vehicle vs. control+vehicle p = 0.007. No significant differences were observed for number of crossings of the analogous, virtual opposite platform location (not shown). (C) 2-way ANOVA with repeated measures revealed significant effects of genotype, F1,20 = 9.91, p = 0.005 and day, F10,200 = 21.42, p = 0.001, as well as a significant interaction between genotype and DAPT, F1,20 = 5.43, p = 0.03. Post-hoc planned comparison test revealed significant differences between Ts65Dn+vehicle vs. all 3 other groups (vs. Ts65Dn+DAPT p = 0.04, vs. control+vehicle p = 0.003, and vs. control+DAPT p = 0.005). (D) 2-way ANOVA with repeated measures revealed main effects of genotype, F1,20 = 5.13, p = 0.03 & day F10,200 = 21.94, p<1.00E-06 with an interaction between genotype and DAPT, F1,20 = 5.43, p = 0.03. Post-hoc planned comparison test revealed only a significant difference between Ts65Dn+vehicle vs. control+vehicle p = 0.004.

Measuring the time it takes to reach the hidden platform during training (latency), Ts65Dn mice treated with vehicle alone exhibited significantly poorer learning compared to control mice treated with vehicle (Fig. 2a), confirming the previously reported cognitive deficits in these mice [19]. Treatment of Ts65Dn mice with DAPT, however, completely reversed these deficits, such that learning in DAPT-treated Ts65Dn mice was not significantly different from learning in control mice treated with vehicle or DAPT (Fig. 2a). Importantly, average daily swim speed was measured throughout the experiment and was not significantly different among any of the groups (data not shown).

Following 11 days of training, a probe trial was performed on day 12 to assess spatial memory. Using the number of times mice crossed the target platform location as a measure, Ts65Dn mice treated with vehicle alone showed a dramatically decreased number of crossings compared to vehicle or DAPT-treated control mice. In contrast, the number of target platform crossings for Ts65Dn mice treated with DAPT was equivalent to those of control mice treated with vehicle or DAPT (Fig. 2b). Neither DAPT nor vehicle significantly affected number of crossings for an arbitrary point in the pool (equivalent target location in opposite quadrant; not shown).

In agreement with previous reports [19], Ts65Dn mice exhibited poorer learning on a visible platform version of the water maze. This deficit was also rescued by treatment with DAPT (Fig. 2c). Interestingly, a slight increase in thigmotaxis (tendency to swim near the walls of the water maze) in the Ts65Dn mice was also reversed by DAPT (Fig. 2d), suggesting the complexity of the cognitive and behavioral phenotype that might be affected by Aβ.

## Discussion

The notion that DS symptoms represent an irreversible developmental defect has been challenged recently by demonstrations that cognition in Ts65Dn mice can be improved pharmacologically using either GABA_A_ antagonists [21], [22], memantine (an NMDA receptor antagonist) [23], or the noradrenergic agonist prodrug, L-DOPS [24]. Together these observations suggest that cognitive improvement in Ts65Dn mice occurs by enhancing or otherwise regulating excitatory synaptic transmission. This is consistent with observations suggesting that GABAergic over-inhibition of excitatory synaptic activity causes loss of synaptic plasticity in Ts65Dn mice [25]. Here we provide evidence that cognitive deficits in DS can be corrected by controlling Aβ production, itself a regulator of glutamatergic transmission. Specifically, we propose that the cognitive improvement we observed in Ts65Dn mice treated with the γ-secretase inhibitor, DAPT, resulted from lowered Aβ levels [26], [27], [28].

Our results contribute to a growing body of evidence that supports the hypothesis that cognitive function undergoes rapid change in response to fluctuations in soluble Aβ levels in cognitively impaired animals. Administration of DAPT to the Alzheimer's mouse model, Tg2576 corrects cognitive deficits characteristic of this strain after exposing the mice to the drug for as little as 3 hours prior to testing [20]. Tg2576 mice express human APP containing the familial Alzheimer's disease (FAD) Swedish mutation, which results in elevated levels of Aβ peptides [29]. Tg2576 mice begin to develop amyloid plaques at about 12+ months. However, soluble Aβ levels are elevated by five months and the mice are cognitively impaired at this time. The rapid correction of the cognitive deficit in these mice, ages 5 to 16 months, by DAPT administered only 3 hours prior to testing coincides with the amount of time required to reduce levels of soluble Aβ by about one half [16].

Other studies have shown that soluble Aβ rapidly inhibits hippocampal long-term potentiation (LTP) *in vivo* [30] and depresses excitatory synaptic transmission in hippocampal slice neurons [31]. A mechanism that would account for these effects of Aβ is suggested by studies in which soluble Aβ has been shown to induce rapid internalization of NMDA- [26] and AMPA-type glutamate receptors [27], [28]. These cellular events suggest a mechanism that could explain reduced cognitive function in the context of Aβ overexpression as well as the rapid recovery of cognitively impaired animals treated with Aβ-lowering drugs.

In human DS, children exhibit intellectual disability prior to the development of a neurodegenerative phenotype or the development of amyloid plaques [9]. This does not rule out developmental abnormalities as contributors to intellectual disability. However, given the rapid amelioration of cognitive deficits in Ts65Dn mice by DAPT, we suggest that intellectual disability in young DS patients might also be treatable by Aβ-lowering drugs.

## Materials and Methods

### Ethics Statement

The care of the animals and sacrifice procedures in this study were performed according to the National Institutes of Health Guide for the Care and Use of Laboratory Animals and were approved by the Institutional Animal Care and Use Committee of The Rockefeller University.

### Mouse lines

All mice were purchased from The Jackson Laboratory (Bar Harbor, ME) and maintained at The Comparative Bioscience Center (CBC) at The Rockefeller University. These consisted of Ts65Dn (trisomic) females and normosomic (disomic) colony-mate females as controls. Females were chosen, rather than males, to facilitate housing (5 to a cage). A previous study demonstrated that male and female Ts65Dn mice share equivalent learning deficits [12]. Although onset of estrus cycle in some Ts65Dn females may be delayed by one to two weeks, by eight weeks of age they are cycling the same as their diploid littermates. Since female mice (in general) when housed together, tend to become synchronous, the Ts65Dn mice would likely be synchronous with same-aged controls or littermates (Personal communication, Muriel T. Davisson, PhD, The Jackson Laboratory). Thus, for a given experiment, the mice were assumed to be in similar phases of the estrus cycle. The methods of breeding, genotyping and other pertinent information can be obtained through the Jackson lab web address: http://www.jax.org/cyto/ts65dn.html. Briefly, Ts65Dn mice (also designated: Ts(17^16^)65Dn) result from crossing Ts65Dn females to C57BL/6JEi×C3H/HeSnJ (B6EiC3Sn) F1 males. Quantitative PCR is used to identify trisomic mice. Normosomic controls consist of wild type mice (not harboring the segmental trisomy mutation) that are derived from the Ts65Dn colony. The recessive retinal degeneration 1 mutation (*Pde6b^rd1^*) segregates in this colony. Progeny are genotyped by standard PCR to screen out all mice harboring this gene.

### Detection of APP and APP CTFs

Mice were sacrificed by CO_2_ asphyxiation and brains were immediately dissected. Cerebellum was removed and a hemibrain (volume approx. 200ml) was homogenized in 600ml of 3% SDS containing protease inhibitor cocktail, then sonicated and heated at 95°C for 10 min, followed by a second round of sonication. The resulting lysates were centrifuged at 13,000×g for 20 minutes at room temperature and supernatants were removed for analysis. After normalizing for protein concentration (bicinchoninic acid method), aliquots of each sample containing approximately 25mg of protein (5ml) were mixed with 20ml SDS sample buffer and resolved by SDS-PAGE on pre-cast 10–20% tricine Novex gels (Invitrogen). After electro-transfer to a PVDF membrane (pore diameter, 0.45mM), western blots were prepared using antibody 369 (which recognizes the C-terminal region of APP and APP-CTFs) or anti-b-actin (Santa Cruz Biotechnology, sc-4778). APP, CTFs and actin were resolved by chemiluminescence on Kodak film. There were four groups of mice: Ts65Dn +/− DAPT, controls +/− DAPT. Each group consisted of 8 animals.

### Detection of mouse Aβ by ELISA

Sandwich Elisa was carried out for endogenous mouse Aβ as previously described [32]. The organic solvent, diethyl amine (DEA), was used to extract soluble Aβ [33]. Briefly, hemi-brains were homogenized in 20 mM Tris buffer containing 1 mM EDTA, 1 mM EGTA, 250 mM sucrose and protease inhibitors, pH 7.4. The lysate was further homogenized with 0.4% DEA in 100 mM NaCl and centrifuged at 135,000×*g* for 60 min. The supernatant was neutralized by adding 0.5 M Tris-HCl, pH 6.8. The ELISA assay was performed as described previously [34]. Briefly, Nunc-immuno plates (Maxisorp; Nunc A/S, Roskilde, Denmark) were coated with 10 µg/ml JRF/cA40/10 or JRF/cA42/26 antibodies. Mouse-specific antibody JRF/A1–15/2-HRPO was used to detect the presence of Aβ peptides. There were four groups of mice: Ts65Dn +/− DAPT, controls +/− DAPT. Each group consisted of 6 animals.

### DAPT administration

(N-[N-(3,5-Difluorophenacetyl-L-alanyl)]-S-phenylglycine *t*-Butyl Ester) was purchased from EMD Biosciences, Inc. and Sigma-Aldrich Co. Formulation and administration were carried out as described [16], [35]. Briefly, DAPT was suspended in 100% ethanol (3mg/0.015ml), which was then rapidly mixed with filter-sterilized Mazola corn oil (10 mg/ml suspension in 5% ethanol/95% corn oil) by vortexing. 150µl was injected s.c. twice daily, and 300µl was injected in a single dose on the first and last day, per 30g mouse. Mice received ∼100mg DAPT/kg/day. This dose was based on the quantity of DAPT reported to effectively lower Aβ levels in mice, while allowing daily administration for up to two weeks without mortality or significant morbidity [16], [35], [36].

### Behavioral testing

Water maze experiments were performed on 4-month-old female Ts65Dn mice and female disomic colony mate controls as previously described except that a single probe trial was conducted on day 12. Visible platform testing, with white vinyl curtains covering external cues, began one day after the probe trial and consisted of 6 trials/day for 3 days. 1.5 mg of DAPT (0.15 ml of a 10 mg/ml suspension in 5% ethanol/95% corn oil) was administered s.c. twice daily 2 days prior to testing and throughout water maze testing. No adverse effects were observed. There were four groups of mice: Ts65Dn +/− DAPT, controls +/− DAPT. Each group consisted of 6 animals. A 1.22 meter diameter, white, plastic, circular pool was filled to a depth of 33 centimeters with 22°C+/−1°C water made opaque with gothic white, non-toxic, liquid tempera paint in a room with prominent extra-maze cues. Mice were placed in one of 4 starting locations facing the pool wall and allowed to swim until finding a 15 centimeter diameter, white platform submerged by 0.75 cm for a maximum of 60 sec. On finding the platform, mice remained on the platform for 20 seconds before being removed to the home cage. If mice did not find the platform within 60 sec, they were guided to the platform by the experimenter and after remaining on the platform for 20 sec were removed to the home cage. Latency to reach the platform, distance traveled to reach the platform, swim speed, time spent in each of 4 quadrants and time spent along the walls were obtained using automated video tracking software from Noldus (Ethovision). Mice were trained with 4 trials/day with an inter-trial interval of 1–1.5 min for 11 consecutive days between 8 AM and 1 PM. A probe trial (free swim with the submerged platform removed) was performed as the first trial of the day on day12. The number of platform location crossings during the probe trial was calculated and analyzed with Student's t-test while latency to platform, swim speed and thigmotaxis (tendency to remain near walls) were analyzed using ANOVA with repeated measures. In separate experiments, a visual cue was attached to the platform and extra-maze cues were covered with white plastic curtains. Latency to reach the visible platform was recorded for 4 different, random platform locations with an intertribal interval of 1 min. The visible platform test examines the animal's gross visual ability.

## Acknowlegments
